# Cost-effectiveness of a mentalisation-based treatment for antisocial personality disorder in males convicted of an offence on community probation in England and Wales: Multicentre, assessor-blind randomised controlled trial

**DOI:** 10.1371/journal.pone.0352865

**Published:** 2026-07-22

**Authors:** Barbara Barrett, Elizabeth Simes, Karen Yirmiya, James M. S. Wason, Alison Frater, Angus Cameron, Stephen Butler, Zoe Hoare, Mary McMurran, Paul Moran, Mike Crawford, Stephen Pilling, Elizabeth Allison, Jessica Yakeley, Anthony Bateman, Peter Fonagy

**Affiliations:** 1 Institute of Psychiatry, Psychology & Neuroscience, King’s College London, United Kingdom; 2 Research Department of Clinical, Educational and Health Psychology, University College London, London, United Kingdom; 3 Anna Freud, London, United Kingdom; 4 Population Health Sciences Institute, Newcastle University, Newcastle upon Tyne, United Kingdom; 5 School of Law, Royal Holloway, University of London, London, United Kingdom; 6 National Probation Service London Division, London, United Kingdom; 7 Psychology Department, University of Prince Edward Island, Charlottetown, Prince Edward Island, Canada; 8 NWORTH Clinical Trials Unit, North Wales Medical School, Bangor University, Gwynedd, United Kingdom; 9 Institute of Mental Health, University of Nottingham, Nottingham, United Kingdom; 10 Centre for Academic Mental Health, Population Health Sciences Department, Bristol Medical School, University of Bristol, Bristol, United Kingdom; 11 Division of Psychiatry, Imperial College London, London, United Kingdom; 12 Portman Clinic, Tavistock and Portman NHS Foundation Trust, London, United Kingdom; University of York Centre for Health Economics, UNITED KINGDOM OF GREAT BRITAIN AND NORTHERN IRELAND

## Abstract

This study addresses a critical gap in the evidence base for cost-effective psychological interventions for antisocial personality disorder (ASPD) in forensic settings by presenting a full trial-based economic evaluation of mentalisation-based treatment plus probation as usual (MBT-ASPD + PAU), compared to PAU alone, for males with ASPD convicted of an offence in England and Wales. The economic evaluation adopted a societal perspective, incorporating costs to health care, the criminal justice system, and the wider societal impact of offending. Outcomes were assessed 24 months post-randomisation. The primary cost–utility analysis compared costs with quality-adjusted life years (QALYs) derived from the EQ-5D-5L, while a secondary cost-effectiveness analysis compared costs with aggression outcomes measured by the Overt Aggression Scale – Modified (OAS-M). Although missing data limited definitive conclusions regarding service use, available data suggest that participants in the PAU group spent more time in custody than those in the MBT-ASPD + PAU group. Utility scores and quality of life showed minimal variation over follow-up, with no significant differences between groups. Overall costs were lower in the MBT-ASPD + PAU group, though not statistically significant. Over the 24-month period, MBT-ASPD + PAU was associated with both reduced costs and lower aggression (as reflected in OAS-M scores), indicating that each unit reduction in aggression yielded a cost saving of approximately £93. For QALYs, MBT-ASPD + PAU appeared cost-effective compared to PAU across all values a decision-maker may be willing to pay for improvements in outcome. These findings provide preliminary evidence for the potential economic value of MBT-ASPD + PAU in a forensic ASPD population, particularly in relation to aggression reduction and the associated cost savings.

**Trial registration**This trial is registered with ISRCTN (ISRCTN 32309003).

## Introduction

Antisocial personality disorder (ASPD) is characterised by a persistent pattern of behaviours including chronic aggression, recklessness, irritability, deceitfulness, irresponsibility, and a marked lack of remorse [1]. Although its prevalence in the general population is relatively low [2], rates can reach up to 50% among men convicted of violent offences, where it is associated with more severe and persistent offending [3]. ASPD imposes a substantial economic burden on both health and criminal justice services in community [4] and forensic settings [5]. Despite this, evidence on the cost-effectiveness of pharmacological or psychological interventions remains extremely limited, constraining efforts to optimise resource allocation. Such evidence is especially important given the high rates of recidivism in individuals with ASPD [6], with significant potential for long-term cost savings across health and criminal justice systems.

The impact of ASPD extends beyond the individual, straining family relationships, disrupting social functioning, and impeding the formation of meaningful connections [7]. From a criminal justice perspective, the disorder contributes to serious harm and enduring consequences for victims [8], while complicating the management of offenders in both custodial and community contexts [9].

Preliminary evidence supports the cost-effectiveness of psychological interventions for personality disorders, including ASPD, in community settings [10,11]. However, to our knowledge, there are no economic evaluations of interventions for ASPD or associated aggression within forensic environments. Current treatment provision remains fragmented and inconsistently delivered, largely due to a lack of robust evidence [12]. The UK Offender Personality Disorder Pathway represents an important attempt to integrate psychologically informed care within the criminal justice system [13,14]. Nevertheless, high-quality evidence on cost-effectiveness is urgently required to guide strategic resource use. In its absence, delivering effective and socially beneficial interventions for individuals with ASPD will remain a major challenge. This study presents a full trial-based economic evaluation of *mentalisation-based treatment* for ASPD in males convicted of an offence in England and Wales.

## Methods

We report the economic evaluation of the *Mentalisation for Offending Adult Males* (MOAM) study, described in detail elsewhere [15]. This multicentre, two-arm pragmatic RCT evaluated the effectiveness of *mentalisation-based treatment* for antisocial personality disorder (MBT-ASPD) combined with *probation as usual* (PAU), compared to PAU alone. Participants were males aged 21 or older, identified through the Offender Personality Disorder Pathway, who met DSM-5 criteria for ASPD and scored at least 15 on the Overt Aggression Scale – Modified (OAS-M). A key inclusion criterion for trial participants was adequate English language and cognitive capacities to participate in informed consent and group therapy. Potential participants were provided with participant information sheets before providing consent. Each site’s lead MBT clinician assessed potential participants’ capacity to provide informed consent and countersigned the consent form if satisfied. Recruitment took place between 1^st^ January 2016 and 31^st^ August 2018. The primary outcome for the effectiveness evaluation was aggression at 12 months post-randomisation, assessed using the OAS-M. Results indicated that mean OAS-M scores at 12 months, as well as total scores over the 24-month trial period, were significantly higher in the PAU group than in the MBT-ASPD group [15]. The study protocol was approved by the London – South East Research Ethics Committee (reference number 14/LO/1696) and the National Offender Management Service (reference number 2014−315).

The economic evaluation assessed costs and outcomes at 24 months post-randomisation. The primary analysis was a cost–utility analysis using quality-adjusted life years (QALYs) derived from the EQ-5D-5L. A secondary cost-effectiveness analysis examined aggression outcomes using the OAS-M.

Although economic evaluations conducted within NHS contexts typically adopt an NHS and Personal Social Services perspective [16], this analysis employed a partial societal perspective. This included costs to health care, social and personal care services, the voluntary sector, criminal justice agencies (including probation), and the economic impact of criminal activity. A broader perspective was justified due to the intervention’s delivery within the criminal justice system and its anticipated cross-sectoral impact.

Building on previous work in this area [17], and in collaboration with team members with both lived and professional experience of prison and probation services, we adapted the Secure Facilities Service Use Schedule (SF-SUS) - a self-report tool that records use of service provided accommodation, education, employment and training, primary and secondary health and social care, criminal justice and medication - for use in this study. The SF-SUS has been used in previous economic evaluation in forensic settings and adapted for use in different populations [18]. Validation is through extensive field use, expert consultation, and iterative refinement rather than through psychometric testing [19], and the measure was co-developed with clinicians and individuals with lived experience of prison and probation environments to ensure content validity, feasibility, and completeness of the included resource use categories. The SF-SUS was administered via structured interviews by trained traditional and lived experience researchers at baseline, and at 6-, 12-, 18-, and 24-month follow-ups. Intervention attendance data were collected through routine therapist reporting to commissioners. Data on criminal activity were obtained from the Police National Computer, as described elsewhere [17].

All costs were calculated in 2018–2019 GBP. Where required, costs from earlier or later years were adjusted using appropriate inflation indices [20]. Costs incurred beyond 12 months were discounted at the NICE-recommended rate of 3.5% [16]. MBT costs were estimated using a detailed micro-costing (bottom-up) approach [20], which included indirect time (e.g., preparation, meetings, supervision, calls) and direct contact time. For group interventions, costs were allocated to all group members, regardless of attendance [21]. Other unit costs were drawn from standard sources [20,22,23], and crime-related costs were based on the most recent UK government data [24], adjusted to exclude response-related costs to avoid double counting with SF-SUS data.

The cost-effectiveness analysis used the primary outcome,frequency of aggressive antisocial behaviour, measured by the OAS-M. The cost–utility analysis used QALYs derived from the EQ-5D-5L, collected at baseline, 6, 12, 18, and 24 months [25]. EQ-5D-5L responses were mapped to the EQ-5D-3L value set using the NICE-recommended crosswalk algorithm [16]. QALYs were calculated assuming a linear trajectory between time points [26], and values beyond 12 months were discounted at 3.5% [16].

We followed best practice for handling missing data in trial-based economic evaluations [27]. An assessment of the extent and structure of missingness supported the assumption that data were missing at random, with predictors of missingness modelled in the imputation procedure. Missing values were therefore imputed using multiple imputation with chained equations and predictive mean matching. The analysis was based on 50 multiply imputed datasets for total costs and outcomes [28] with the following variables included in the model: age, baseline cost baseline OAS-M scores, and type and sentence length for the index offence. The number of participants with available cost and outcome data by trial arm and time point was reported, along with reasons for non-response and baseline predictors of missingness. All analysis was completed in STATA 18.

Service use and cost differences between arms were described using means and standard deviations; no statistical significance testing was conducted. Because cost data are often skewed, bootstrapped confidence intervals were calculated in a complete case analysis to explore non-normality [29]. Mean total costs at 24 months were compared using linear regression models, adjusting for baseline costs, baseline utility, baseline OAS-M scores, sentence length for the index offence, and randomisation stratifiers. Summaries of costs and utilities are presented using complete case data to transparently present observed service use patterns.

Costs and outcomes were considered jointly in both the cost-effectiveness and cost–utility analyses. Incremental cost-effectiveness ratios (ICERs) were calculated, and uncertainty around these estimates was examined using bootstrapped cost-effectiveness planes and cost-effectiveness acceptability curves (CEACs) [30]. For the QALY-based CEAC, willingness-to-pay thresholds ranged from £0 to £30,000. For the OAS-M analysis, given limited prior use of CEACs with this outcome, the willingness-to-pay range was extended from £0–£3,000 (as in the only prior study) to £0–£5,000 [31].

## Results

The pattern of missingness was complex, reflecting the inherent challenges of maintaining follow-up over a two-year period in this population. Baseline SF-SUS data were complete; however, completion rates dropped substantially at the 12- and 24-month follow-ups. In many instances, participants completed the SF-SUS at either 12 or 24 months, resulting in partial follow-up data for a large proportion of the sample. Only 17% of participants completed SF-SUS at all follow-up points (Table 1). Baseline characteristics of those with and without complete data are presented in Table 2. Participants with complete data were slightly older, more likely to be recruited from specific sites, and more often had fewer than 12 months remaining on their sentence. Differences by ethnicity were difficult to interpret due to small subgroup sizes. Crucially, data completion rates were comparable across trial arms.

**Table 1:** Availability of service use and EQ5D-5L data at baseline and each follow-up.

Time point	N available	N missing	% missing
Baseline SF-SUS	312	1	0
12M SF-SUS	95	218	70
24M SF-SUS	107	206	66
TOTAL SF-SUS (12 + 24M)	52	261	83
Baseline utility	305	8	3
12M utility	140	173	55
24M utility	204	109	35
Baseline OASM	309	4	1
12M OASM	108	205	65
24M OASM	156	157	50

**Table 2:** Percentage (%) of participants with full SF-SUS data by baseline characteristics.

	Some missing data, mean or n(%)		full data, mean or n(%)	
**Age**	34.35		37.05	
**Group**				
PAU	133	(85%)	23	(15%)
PAU + MBT	127	(81%)	30	(19%)
**Site**				
site 1	18	(95%)	1	(5%)
site 2	17	(85%)	3	(15%)
site 3	18	(100%)	0	(0%)
site 4	24	(100%)	0	(0%)
site 5	13	(59%)	9	(41%)
site 6	17	(68%)	8	(32%)
site 7	28	(68%)	13	(32%)
site 8	16	(94%)	1	(6%)
site 9	23	(100%)	0	(0%)
site 10	12	(71%)	5	(29%)
site 11	24	(86%)	4	(14%)
site 12	21	(84%)	4	(16%)
site 13	29	(85%)	5	(15%)
**Time remaining sentence**				
12 months or more	170	(82%)	38	(18%)
less than 12 months	38	(72%)	15	(28%)
**Ethnicity**				
White British	192	(82%)	41	(18%)
White Irish	5	(83%)	1	(17%)
White Other	7	(88%)	1	(13%)
Mixed – White and Black Caribbean	14	(74%)	5	(26%)
Mixed – White and Black African	2	(100%)	0	(0%)
Mixed – White and Asian	2	(67%)	1	(33%)
Mixed – other	4	(80%)	1	(20%)
Black British Caribbean	19	(90%)	2	(10%)
Black British African	2	(100%)	0	(0%)
Black British Other	7	(100%)	0	(0%)
Asian British Indian	1	(50%)	1	(50%)
Asian British Pakistani	1	(100%)	0	(0%)
Asian British Bengali	2	(100%)	0	(0%)
Asian British Other	1	(100%)	0	(0%)
**Marital Status**				
never married	220	(85%)	40	(15%)
married	6	(100%)	0	(0%)
separated	5	(56%)	4	(44%)
divorced or annulled	12	(63%)	7	(37%)
engaged	2	(67%)	1	(33%)
live-in partner	14	(93%)	1	(7%)

On this basis, we found no systematic pattern in the missing data, supporting the assumption that data were missing at random and justifying the use of multiple imputation. While service use and cost data are presented using a complete case analysis, the primary cost-effectiveness analyses were conducted on the multiply imputed dataset.

Service use over the 24-month follow-up suggested that participants in the PAU group spent more time in custody (mean: 6.96 months, SD: 7.59) compared to those in the MBT-ASPD + PAU group (mean: 4.54 months, SD: 7.03). Use of health and social care services, including medication, was broadly similar across groups (Table 3).

**Table 3:** Service use by randomised group over 24 months follow-up, complete case.

	TAU (n = 23)			MBT (n = 30)		
	Mean	SD	% using	Mean	SD	% using
**Accommodation**						
Private home	4	5.99	43.00	2.72	5.37	30.00
Private rental	4.48	7.25	48.00	7	7.20	67.00
LA/HA rental	4.04	5.96	43.00	5.04	7.27	45.00
AP/bail hostel	1.26	2.88	27.00	1.82	4.23	24.00
B&B/boarding house	0.26	1.25	4.00	0.2	1.10	3.00
Hostel day staff	1.09	3.03	13.00	1.57	3.79	23.00
Hostel 24h staff	0.96	2.64	22.00	0.6	1.67	13.00
Prison/YOI	6.96	7.59	57.00	4.54	7.03	40.00
Living on streets	0.3	1.46	4.00	0	0.00	0.00
**Health and social care services**						
GP prison	6.48	19.63	48.00	0.97	1.90	27.00
GP community	7.13	8.80	91.00	7.90	7.51	87.00
Nurse prison	2.96	5.38	43.00	25.53	101.79	33.00
Nurse community	1.39	1.70	61.00	3.23	5.35	73.00
Mental health nurse prison	3.65	14.19	22.00	0.40	1.30	13.00
Mental health nurse community	8.65	18.39	61.00	16.53	39.05	43.00
Counsellor prison	4.30	12.13	22.00	0.93	3.08	20.00
Counsellor community	5.57	15.30	35.00	5.00	17.30	27.00
Psychiatrist prison	1.96	5.68	30.00	0.37	1.38	10.00
Psychiatrist community	1.30	4.40	22.00	1.27	4.89	13.00
Smoking cessation prison	1.17	3.07	22.00	0.07	0.37	3.00
Smoking cessation community	0.22	0.85	9.00	0.43	2.01	10.00
Social worker prison	0.04	0.21	4.00	0.00	0.00	0.00
Social worker community	5.74	17.28	26.00	5.20	15.20	27.00
Addiction support worker prison	2.35	4.43	35.00	5.63	19.42	30.00
Addiction support worker community	3.35	4.67	61.00	5.97	11.32	40.00
Advice worker prison	0.57	1.47	17.00	0.07	0.02	9.00
Advice worker community	3.87	7.38	57.00	2.10	3.36	47.00
Employment advisor prison	0.35	1.07	17.00	0.23	0.63	17.00
Employment advisor community	2.65	5.16	35.00	4.10	8.20	37.00
**Hospital services**						
Inpatient physical health prison	1.04	5.00	4.00	0.00	0.00	0.00
Inpatient physical health community	0.48	1.34	13.00	4.67	18.63	40.00
Inpatient mental health prison	0.04	0.21	4.00	0.00	0.00	0.00
Inpatient mental health community	0.13	0.63	4.00	2.33	12.78	3.00
Outpatient physical health prison	0.52	1.04	30.00	0.10	0.31	10.00
Outpatient physical health community	3.17	6.75	57.00	10.73	39.78	60.00
Outpatient mental health prison	0.04	0.21	4.00	0.00	0.00	0.00
Outpatient mental health community	1.30	6.04	9.00	0.10	0.31	10.00
A&E prison	0.48	1.08	30.00	0.10	0.31	10.00
A&E community	1.83	2.48	61.00	2.40	4.19	53.00
Ambulance prison	0.13	0.46	9.00	0.00	0.00	0.00
Ambulance community	0.39	1.12	17.00	2.13	4.90	5.00
Probation prison	1.35	3.20	30.00	3.27	8.19	37.00
Probation community	44.35	32.67	96.00	42.73	22.02	97.00
Probation group prison	0.30	1.46	4.00	0.30	1.47	7.00
Probation group community	5.48	12.90	35.00	7.40	14.92	30.00
Solicitor prison	2.35	5.47	35.00	2.27	5.51	37.00
Solicitor community	8.57	26.89	65.00	2.17	4.40	43.00
Police non arrest prison	0.17	0.65	9.00	0.53	2.57	7.00
Police non arrest community	5.65	9.30	52.00	2.97	9.42	53.00
Police arrest prison	0.40	0.21	4.00	0.07	0.37	3.00
Police arrest community	1.78	1.93	61.00	0.57	0.82	40.00
Police custody prison	0.13	0.63	4.00	0.13	0.57	7.00
Police custody community	2.04	3.02	57.00	0.50	0.78	37.00
Sleeping tablet			36.00			23.00
Anti-anxiety			36.00			43.00
Anti-psychotic			13.00			13.00
Anti-mania			0.00			10.00
ADHD			9.00			13.00
Antidepressant			17.00			23.00

EQ-5D-5L utility scores and derived QALYs are summarised in Table 4. Utility scores remained relatively stable across time points, with minimal variation in quality of life and no statistically significant group differences.

**Table 4:** Mean utility scores and QALYs per participant: complete case.

	MBT-ASPD + PAU			PAU			MBT minus PAU			
	Mean	SD	N	Mean	SD	N	Unadjusted difference^a^	Adjusted difference^b^	95% CI^bc^	p-value
Baseline utility	0.72	0.299	154	0.671	0.298	151				
6 months utility	0.702	0.295	92	0.751	0.275	85				
12 months utility	0.687	0.359	73	0.686	0.271	67				
18 months utility	0.699	0.296	70	0.686	0.263	59				
24 months utility	0.746	0.274	108	0.721	0.292	96				
QALYs over 24 months	1.462	0.453	107	1.378	0.473	92	0.084	0.006	−0.088 to 0.100	0.900

^a^Based on complete case data, ^b^Adjusted for baseline utility, baseline cost, baseline OASM, length of sentence for index offense, time on left on license, age, type of supervision

In the complete case analysis (Table 5), the average cost of the MBT intervention was £826.30 per participant. In the PAU group, the average cost was £42.92, reflecting limited exposure to MBT. Total service costs averaged £34,731.36 in the MBT-ASPD + PAU group and £36,037.09 in the PAU group. Offence-related costs were also lower in the MBT-ASPD + PAU group (£3,371.33) compared to the PAU group (£4,102.10), resulting in overall cost savings of £2,036.50 for the intervention group, although these differences were not statistically significant.

**Table 5:** Mean costs (£) per participant over 24 months follow-up: complete case.

	MBT-ASPD + PAU (n = 30)		PAU (n = 23)		MBT minus PAU			
	Mean	SD	Mean	SD	Unadjusted difference^a^	Adjusted difference^b^	95% CI^bc^	p-value
Baseline	12648.94	5756.40	9708.41	6606.69	2940.53			
Service costs	34731.36	27844.3	36037.09	21267.13	−1305.73	−2160	−18744.57 to 14424.57	0.799
Intervention	826.30	488.01	11.48	42.92				
Health and social care	13054.59	23631.69	8928.12	13346.6				
Criminal justice system costs	20850.48	20079.74	27097.49	16702.36				
Offence costs	3371.33	6654.14	4102.10	4695.78	−730.77	667	−3010.41 to 4345.30	0.731
Total cost	38102.69	28653.62	40139.19	21675.82	−2036.50	−1492.55	−18520.15 to 15535.05	0.864

Given the extent of missing data, the primary cost–utility and cost-effectiveness analyses were conducted using the multiply imputed dataset. For the OAS-M based cost-effectiveness analysis MBT-ASPD was dominant, with lower costs, and lower OAS-M scores, yielding an ICER of £–105.78 and indicating a cost saving of £105.78 per one-point reduction in OAS-M score. In the cost–utility analysis, although costs were also lower in the intervention group, QALYs were marginally worse, resulting in an ICER of £884,308 per QALY. Bootstrapped cost-effectiveness planes and CEACs were generated from the imputed dataset to quantify uncertainty around the cost and outcome estimates.

**Fig 1:**
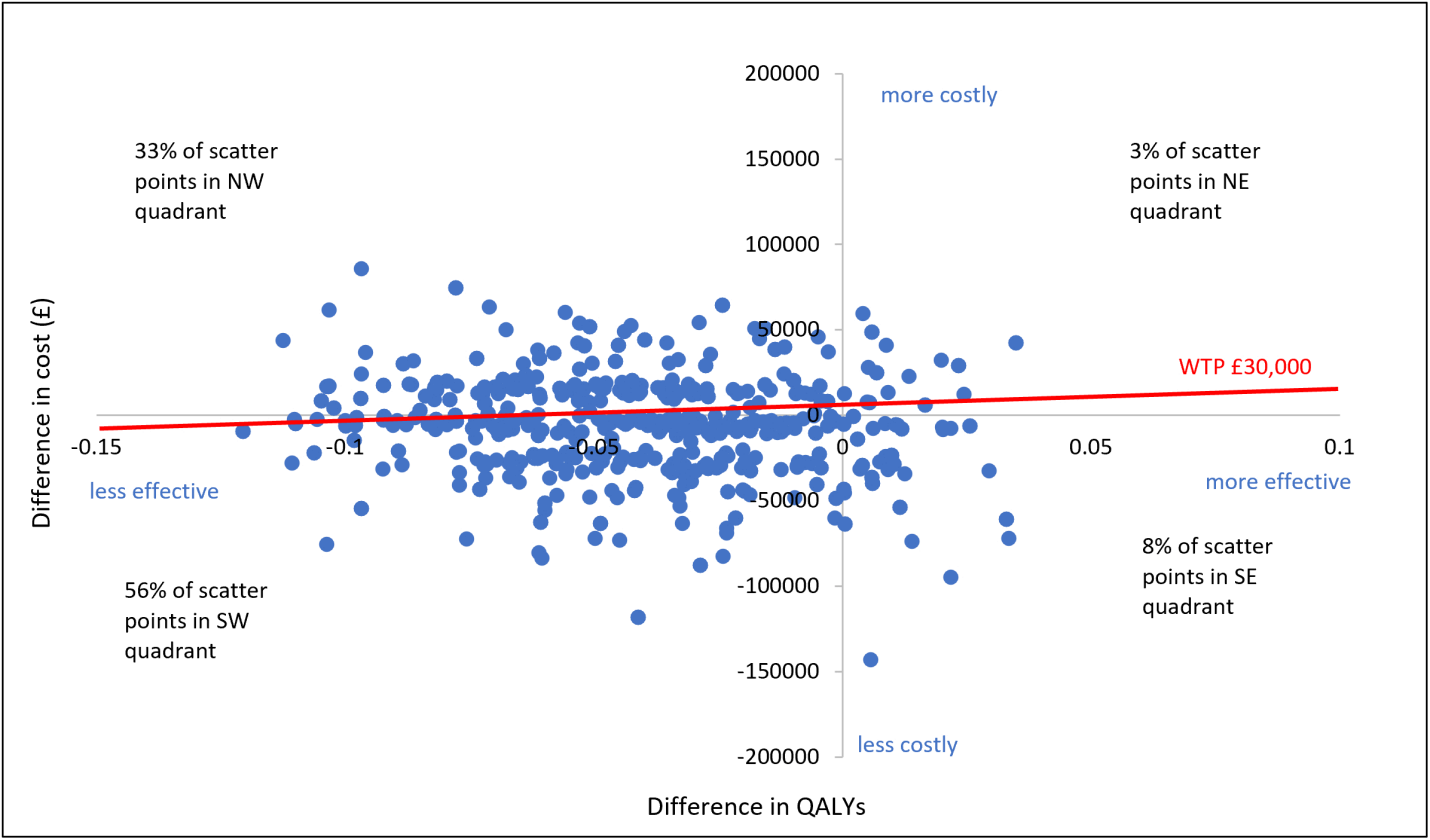
Bootstrapped mean differences in costs and QALYs over 24 months follow-up: multiple imputation.

**Fig 2:**
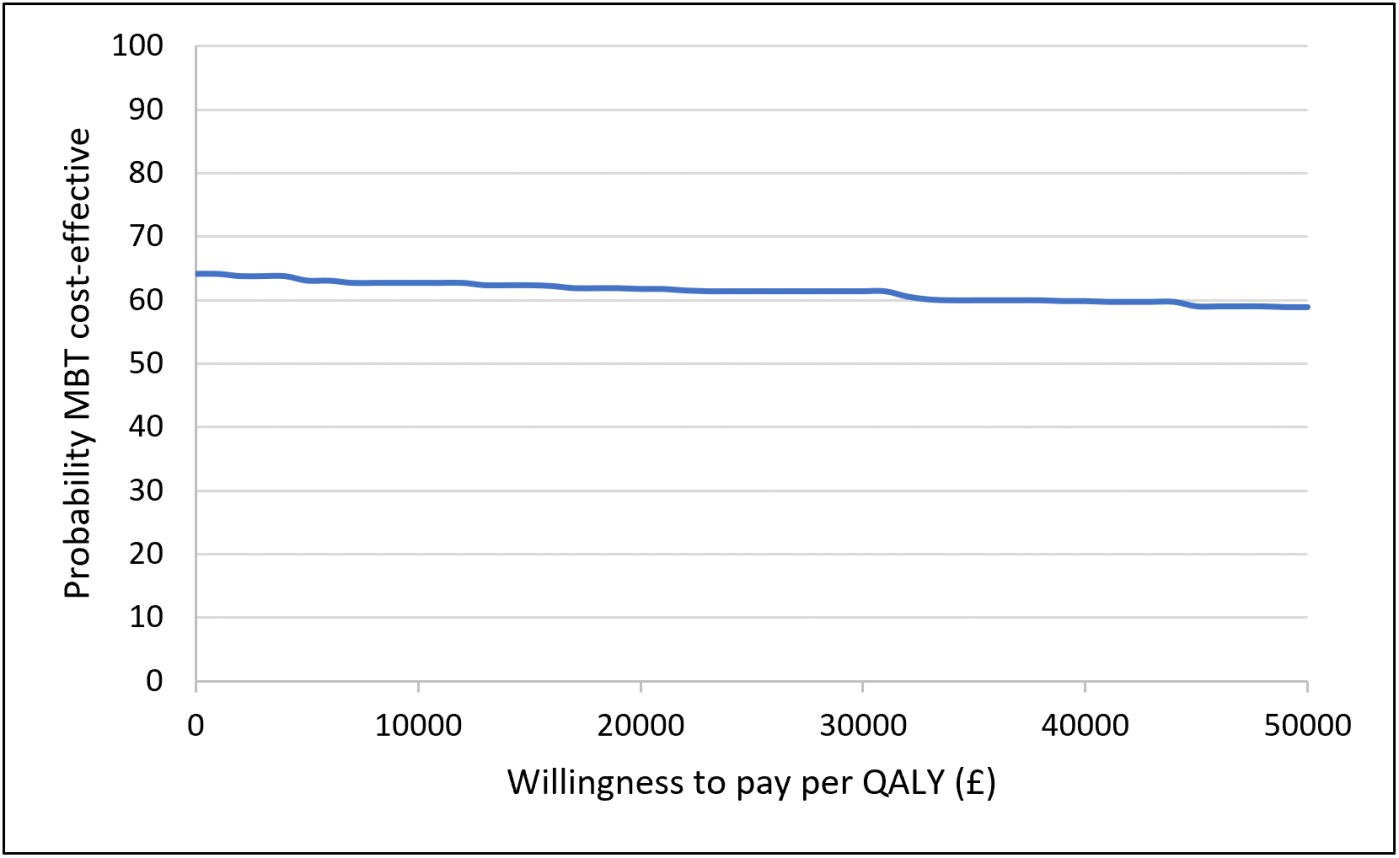
Cost-effectiveness acceptability curve showing the probability that MBT-ASPD + PAU is cost-effective compared with PAU at different values of WTP thresholds per QALY at 24 months follow-up: multiple imputation.

The cost-effectiveness plane for the QALY-based analysis, (Fig 1 Bootstrapped mean differences in costs and QALYs over 24 months follow-up: multiple imputation) presents 1,000 bootstrapped replications of incremental costs and QALYs. Given the minimal difference in QALYs, replications were widely dispersed, with 64% falling in the lower-cost, worse-outcome quadrant. As most replications lay below the £30,000 per QALY threshold (indicated in red), the CEAC (Fig 2 Cost-effectiveness acceptability curve showing the probability that MBT-ASPD + PAU is cost-effective compared with PAU at different values of WTP thresholds per QALY at 24 months follow-up: multiple imputation.) reflects the valuation placed on avoiding QALY losses relative to cost savings and can be considered a willingness to accept. It illustrates how the probability of cost-effectiveness varies across different assumptions about acceptable trade-offs between cost savings and small losses in health.

**Fig 3:**
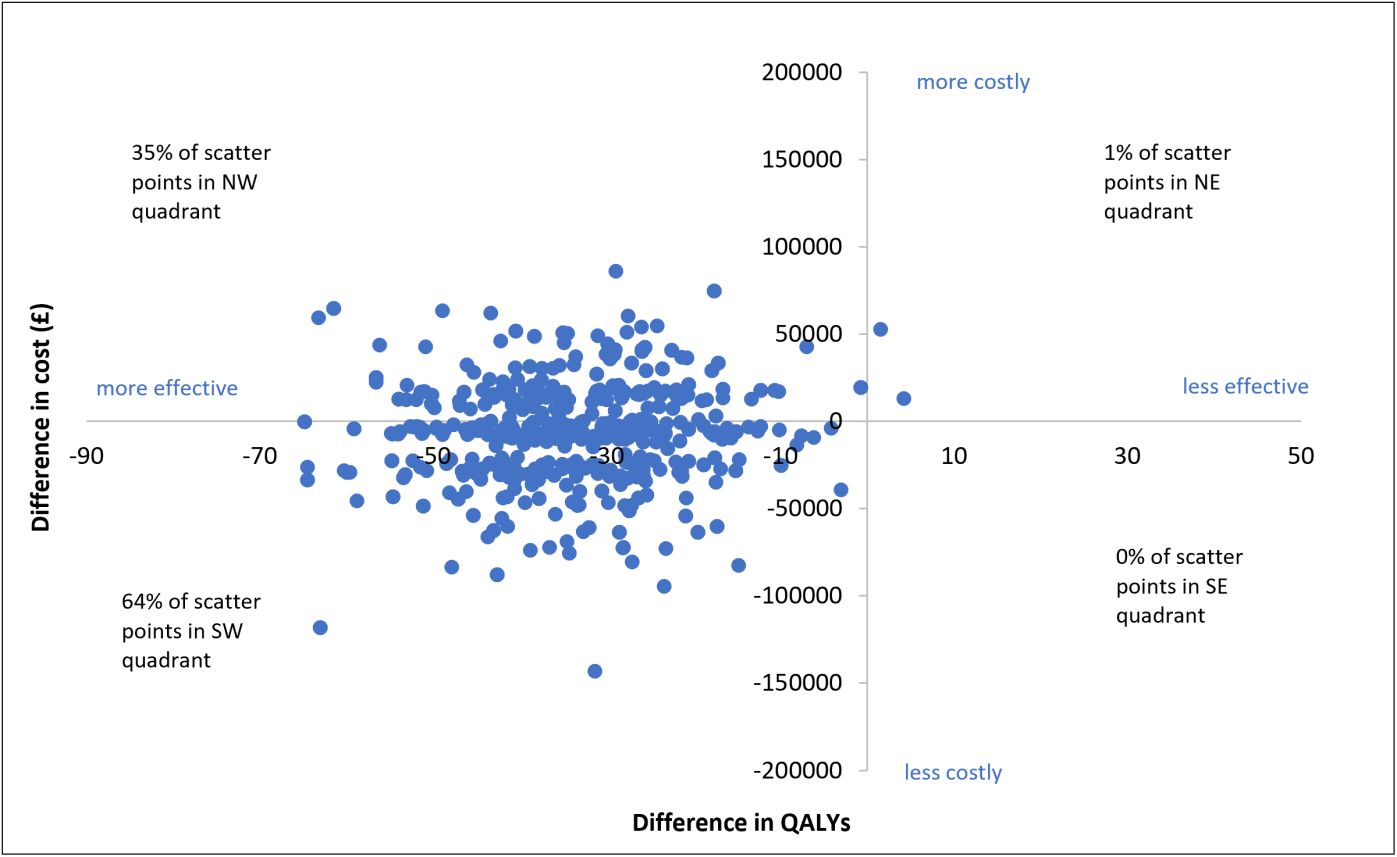
Bootstrapped mean differences in costs and OASM score over 24 months follow-up: multiple imputation.

**Fig 4:**
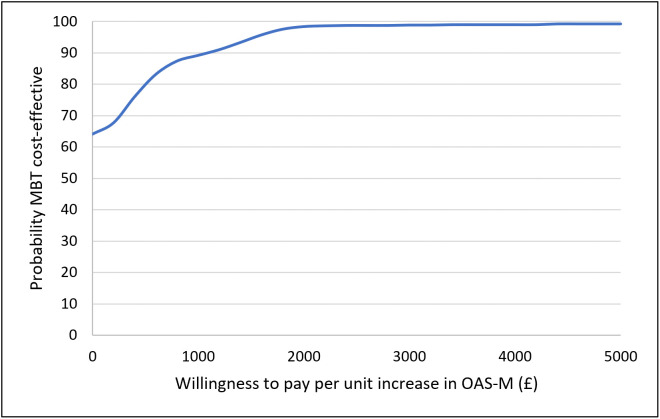
Cost-effectiveness acceptability curve showing the probability that MBT-ASPD + PAU is cost-effective compared with PAU at different values of WTP thresholds per OASM at 34-week follow-up: multiple imputation.

In the OAS-M-based cost-effectiveness analysis (Fig 3 Bootstrapped mean differences in costs and OASM score over 24 months follow-up: multiple imputation.), lower OAS-M scores indicate reduced aggression therefore the x-axis was reversed so that movement to the left reflects improved outcomes. The lower left quadrant represents dominance (lower costs and lower aggression). Here, 99% of bootstrapped replications lay to the left, indicating improved aggression outcomes in the MBT-ASPD + PAU group. Of these, 35% were associated with higher costs (above the x-axis), and 64% with lower costs (below the x-axis), suggesting dominant cost-effectiveness in the majority of replications. Whilst Fig 3 illustrates the joint uncertainty in costs and aggression outcomes, the corresponding CEAC (Fig 4 Cost-effectiveness acceptability curve showing the probability that MBT-ASPD + PAU is cost-effective compared with PAU at different values of WTP thresholds per OASM at 34-week follow-up: multiple imputation) provides the primary basis for decision-making, indicating that even at a willingness to pay of £0 per unit improvement in OAS-M, the probability of MBT-ASPD + PAU being cost-effective was at least 64%, increasing to nearly 100% at approximately £2,000 per unit gain.

Sensitivity analyses, which increased the MBT intervention cost by 50% and included the multiply imputed data resulted in a cost difference of -£1629.18 in favour of MBT-ASPD + PAU, not altering the direction of the cost differences. Similarly, taking a service cost only perspective (health, social care, and criminal justice services) and excluding the costs of offences, resulted in a cost difference of -£4045.56 in favour of MBT-ASPD + PAU, supporting the robustness of the base case findings.

## Discussion

The findings from the imputed analyses suggest a high likelihood that MBT is a cost-effective intervention for individuals with ASPD. The cost of delivering MBT is comparable to other community-based psychological interventions [10], and this expenditure was offset by savings elsewhere, particularly in reduced custodial time and lower offence-related costs. These offence-related savings include reductions in the costs to victims and can therefore be interpreted as reductions in harm. Although overall cost differences were not statistically significant, they informed both the cost–utility and cost-effectiveness analyses, which consistently support the economic case for MBT.

The cost–utility analysis warrants cautious interpretation. Although costs were lower in the MBT-ASPD + PAU group, QALYs were marginally worse, with a mean difference of --0.01, and most bootstrap replications falling in the south-west quadrant of the cost-effectiveness plane. This indicates cost savings accompanied by a small decrease in health related quality of life, the observed difference in QALYs is small and below any meaningful threshold for detectable change, particularly given the complexity of participants’ lives and the broad range of factors that can influence health-related quality of life. Previous research has shown that quality of life is significantly affected by the prison environment [32], which may have diluted the observable impact of the intervention. As has been widely noted, when cost and outcome differences move in opposite directions and are small, the ICER becomes unstable and potentially misleading [33]. In such cases, interpretation is better guided by the cost-effectiveness plane and CEAC. The CEAC does not reflect a willingness to pay for QALY gains, but rather the implicit valuation placed on avoiding small QALY losses relative to achieving cost savings, and can be interpreted as a willingness-to-accept framework. While cost differences were not statistically significant, the direction and consistency of cost reductions across multiple domains, including custodial time and offence-related harms, are highly relevant from a decision-making perspective. In economic evaluations, the focus is not solely on statistical significance but on whether an intervention offers good value for money given the uncertainty around estimates.

The cost-effectiveness analysis based on OAS-M presents a clearer picture. MBT-ASPD + PAU was associated with both lower costs and reduced levels of aggression, resulting in a negative ICER that represents cost savings per unit improvement in OAS-M score. Whilst we report an incremental cost per one point reduction in OAS-M score, it should be noted that this outcome does not have an established minimally important difference, and individual point changes are not easy to interpret. However, this cost-effectiveness analysis, illustrated in both the cost-effectiveness plane and CEAC indicate strong economic dominance of MBT-ASPD + PAU over PAU alone, across the full range of willingness-to-pay thresholds, including zero. The fact that MBT reduced time in custody and offence-related costs suggests that it may offset spending in some of the most resource-intensive areas of public expenditure. From a public sector budgeting perspective, these are precisely the domains where modest behavioural changes can yield substantial cost avoidance.

The principal limitation of this evaluation is the extent of missing data, unsurprising given the high-risk population and extended follow-up period. Nevertheless, the missingness was assessed as random, justifying the use of multiple imputation. The direction of the findings remained unchanged across imputed analyses, although overall cost estimates were higher. Another limitation relates to reliance on participant recall for service use data, which introduces the potential for inaccuracy. However, SF-SUS was administered at 6-month intervals to minimise recall bias, and the fragmented nature of administrative records across criminal justice and health systems meant self-report was the only feasible option. A further limitation is the limited sensitivity of the EQ-5D-5L in this population, which likely constrained the utility-based analysis and its generalisability. The EQ-5D-5L may be poorly attuned to the forms of psychosocial change most relevant to forensic and marginalised populations. As such, the economic value of MBT may be underestimated by conventional cost–utility approaches. Indeed, while our analysis did not quantify return on investment, reductions in reoffending and custodial time imply broader societal benefits, including increased community safety and reduced intergenerational impact, which strengthen the public value proposition of MBT.

A key strength of the study is its two-year follow-up, which allowed the longer-term effects of a behavioural intervention in a forensic population to be captured. The analysis also adopted a broad societal perspective, encompassing costs across health, social care, criminal justice, and victim-related domains, which is an essential approach when evaluating complex interventions with multi-sectoral impacts. The utility of this finding depends, however, on effective integration between health and criminal justice services, which remains inconsistent in practice [34], and full implementation will be required to realise the potential cost-effectiveness of MBT. Despite the substantial missing data, the consistency of findings across complete case, imputed, and sensitivity analyses reinforces the robustness of the conclusion that MBT-ASPD + PAU is a promising, cost-effective intervention for this population. Given its comparability in cost to other community psychological treatments, MBT represents a potentially scalable addition to probation-based services. Its delivery within existing infrastructure, without requiring new capital investment, further supports its feasibility from a public sector commissioning perspective.
