# Supplementary material for: Cost-effectiveness of a mentalisation-based treatment for antisocial personality disorder in males convicted of an offence on community probation in England and Wales: Multicentre, assessor-blind randomised controlled trial
Source: PLoS One. 2026 Jul 22;21(7):e0352865. doi: 10.1371/journal.pone.0352865 (PMC13390880; doi:10.1371/journal.pone.0352865)
Supplement: Attachment — Submitted filename: moam cea plos one response to reviewers 29012026.docx [file pone.0352865.s002.docx]

Review Comments to the Author

Reviewer #1: This manuscript is concise, generally well-written and compelling. The authors describe a cost-effectiveness comparison of probation vs. probation + MBT for men with antisocial personality disorder. They temper their conclusion appropriately given study limitations but suggest that MBT may reduce aggressive behavior and save money. I have a few concerns that are likely addressable via revision.
1. The authors note that they developed the SF-SUS developed for this study. Given the novelty of this measure, I find the lack of psychometric data unsatisfying.

*Thank you for raising this point. Many measurement tools undergo psychometric validation; however, service‑use schedules used in economic evaluations differ conceptually from psychological scales and they are therefore typically validated through developmental refinement and extensive field use, which has been the case with The Secure Facilities Service Use Schedule (SF‑SUS). was adapted for this study from the original Service Use Schedule, which has been used widely in economic evaluations across health and forensic settings. Its validity rests on extensive previous use in large clinical and forensic trials, iterative development and expert feedback, and alignment with established costing frameworks used in UK health economics. This approach is entirely consistent with best practice guidance for economic evaluations in pragmatic trials. To clarify this point, we have added text in the Methods.*

2. Authors report “no systematic pattern in the missing data” but also report that those with complete data were older. Is there no concern that this older sample might be less likely to recidivate or less aggressive? If using their values to predict and impute missing values, might this systematically skew the results?

*Thank you for raising this important point. Our statement that there was “no systematic pattern” refers to the fact that missingness did not vary meaningfully by trial arm and was not associated with baseline aggression or other key outcome‑related variables. The slight age difference you note does not contradict the assumption that the data were missing at random (MAR). We included age in the imputation model, the pattern of missingness was balanced across trial arms, and the findings were consistent across complete case, MI, and sensitivity analyses.*

*We have now clarified this point in the revised manuscript by explaining that observed predictors of missingness (including age) were explicitly modelled in the imputation procedure, thereby minimising the potential for systematic bias.*

3. The authors indicate “a cost saving of £92.69 per one-point reduction in OAS-M score.” I’d like to see more interpretation of this finding – how meaningful is a one-point reduction on the OAS-M?

*We agree that a one-point change on the OAS-M does not have a direct clinical interpretation. We included this CEA as a complementary analysis to the main CUA, and to provide a broader picture of how cost differences relate to outcome measures. We have revised the manuscript to clarify that the cost-effectiveness analysis is best interpreted as an indicator of direction and dominance rather than as a precise valuation of a unit of change.*

Reviewer #2: I’ve read with interest the manuscript “Cost-effectiveness of a mentalisation-based treatment for antisocial personality disorder in males convicted of an offence on community probation in England and Wales : multicentre assessor blind randomised controlled trial. It was nice to read and overall the economic evaluation has been conducted well although some additional analyses need to be added and/or better explained. Please find my remarks below:


Methods:
• The authors mention the Secure Service Use Schedule tool which is a self-reported tool and administered via structured interviews by researchers.
1) What exactly does this tool measure? Please explain this a bit more for readers that are not familiar with the tool.

*Thank you for this helpful suggestion, we have amended the manuscript to describe the different types of service use covered by the SF=SUS.*

• Why were 30 datasets used for multiple imputation? Given that the percentage of missing data exceeds 50%, could you provide a rationale for choosing 30 imputations.

*Thanks for identifying this, the reviewer is correct that the guidance states that the number of imputed datasets should be similar to the percentage of incomplete cases (Faria, 2014) and therefore we have re-run the MI with 50 imputations. The methods section of the paper has been amended to reflect this change, and the results show the revised MI.*

• Lines 151–153: you state that regression analysis was used to compare costs, but it is unclear which type of regression analysis was applied. In addition, based on Table 5, it appears that you also adjusted for baseline utility, but this is not mentioned here. Personally, I am used to applying a seemingly unrelated regression (SUR) to both costs and utility and subsequently using this as input for the economic evaluation. However, it is unclear what exact approach was used in your analysis. Please clarify more.

*We have clarified that we used linear regression here and noted that baseline utility was included in the adjustment terms.*

• The authors present costs and utility scores separately based on complete-case analyses and then only use the multiple imputation (MI) dataset when combining the two outcomes. My suggestion would be to present costs and utility scores based on the MI dataset, as this is standard practice. One option would be to subsequently perform the cost-effectiveness analysis on complete cases and present this as a scenario analysis. This would allow you to discuss the potential impact of using MI versus complete-case analyses on the results and conclusions.

*We thank the reviewer for this thoughtful suggestion. Our decision to present cost and utility scores based on complete data, with MI for the cost-utility and cost-effectiveness analysis, was deliberate. The complete case tables for costs and utility scores are intended to be descriptive, allowing readers to see the observed data that underpin the analysis. We hope that this maintains transparency in our presentation of the trial data. We fully agree that MI is appropriate for the estimation of incremental costs and outcomes, where bias due to missingness is of greatest consequence.*

*We have amended the manuscript to explain our rationale more clearly.*

• It is unclear which variables were included in the imputation model ( like age or other patient characteristics?) Could you please add this info?

*Thanks for the suggestion, this has been added.*

• It is also unclear which software was used for the analysis? Did they use R ( a useful tutorial for a trial based economic evaluation has been published. See: https://doi.org/10.1007/s40273-023-01301-7) or Stata combined with excell? Please specify this in your method section.

*Thanks for the suggestion, this has been added.*

Results
• The ICER based on the OAS-M scores indicates dominance, i.e. lower costs and higher effectiveness. The ICER based on QALYs indicates cost savings but with a QALY loss. I would describe the results explicitly in this way, as this accurately reflects your findings.

*I am not sure why this result is described as difficult to interpret, as it is essentially the mirror image of the quadrant where additional costs are incurred for a QALY gain. The main issue is that a threshold is typically defined for that quadrant but not for the opposite one. For the south-west quadrant, the relevant question is therefore: what do we consider to be minimally acceptable cost savings for a QALY loss? This could be addressed in the Discussion.*

Regardless of whether cost savings per QALY lost or a net benefit approach is used, it always requires a discussion of what level of cost savings might be considered acceptable.

*Thank you for this helpful and constructive suggestion, we agree that the results can be described more explicitly and consistently. We have revised the manuscript to clearly state that the OAS-M based CEA indicates dominance and the QALY based analysis indicates lower costs accompanied by a small QALY loss. Indeed the challenge with the QALY result is not technical (we have removed this) but the absence of an explicit decision threshold for the south west quadrant. We have therefore expanded the discussion section to explicit state this and consider the implications.*

• In addition, given that 56% of the bootstrapped cost–effectiveness pairs lie in the quadrant showing cost savings with a QALY loss, and 33% lie in the north-west quadrant where there are higher costs and a QALY loss (i.e. the intervention is inferior), it is unclear what the willingness-to-pay for a QALY represents in Figure 2. What exactly is being paid for, or should this be interpreted as a willingness to accept rather than a willingness to pay?

*We thank the reviewer for this important observation and agree that the interpretation of the WTP requires clarification. We have revised the text accompanying figure 2 to make it clear that the CEAC illustrates the probability of cost-effectiveness for different values a decision maker is willing to accept in terms of cost savings for small losses in quality of life.*

• Although I understand why the description of the cost-effectiveness plane in Figure 3 was changed, this has become confusing. By switching the axes , it is still not immediately clear that the dominant quadrant reflects an intervention that is more effective and less costly. I am not sure how this could best be resolved. One option might be to only present the cost-effectiveness acceptability curve, because from that figure (please replace “XX” with the figure number) it becomes clear that even under an extreme scenario in which decision-makers are not willing to invest anything, the intervention still has a high probability of being cost-effective.

*Thank you for highlighting this issue; we agree that reversing the axis for the OAS-M based CE plane, whilst analytically correct, may result in confusion. We would prefer to retain figure 3, since it provides a visual representation of the joint distribution of the cost and outcomes and is consistent with the QALY results. We hope to mitigate this by more explicit labelling of quadrants and the figure itself, and cross referencing to the CEAC.*

• Lines 215–217: you mention a sensitivity analysis, but the results are not presented. These should be reported. In addition, it might be useful to add a scenario analysis that only includes healthcare costs, in order to assess the impact of adopting a different analytical perspective.

*We have added in the sensitivity analyses to the results section, and included a service perspective which includes health, social care, and criminal justice costs. We fell that given the setting and the patient group this is a more appropriate perspective that just a health care alone.*

Discussion
• Returning to the interpretation of the results, particularly in line 239: what does it mean that the cost savings exceed plausible thresholds for willingness to pay for a small QALY loss? Why would society ever be willing to pay for a loss in QALYs, or do you mean something else here? This point needs clarification.

*Thank you for identifying this point of potential confusion, we have removed it as (thanks to your earlier comment) we hope this section is now better explained.*

• For the remainder of the Discussion, the authors appropriately describe the limitations of the study and discuss the known issues associated with using a questionnaire such as the EQ-5D-5L in this population. I therefore have no further comments on this section. However, if the additional scenario and sensitivity analyses are conducted, a brief discussion of their implications would be appropriate.

*Thanks for all your very helpful comments. Since the (now) reported sensitivity analyses did not alter the direction or magnitude of the cost differences, and that they are consistent with those already mentioned in the manuscript, we don’t think it’s necessary to add anything additional to the discussion section.*
