# Supplementary material for: Cost-effectiveness of a mentalisation-based treatment for antisocial personality disorder in males convicted of an offence on community probation in England and Wales: Multicentre, assessor-blind randomised controlled trial
Source: PLoS One. 2026 Jul 22;21(7):e0352865. doi: 10.1371/journal.pone.0352865 (PMC13390880; doi:10.1371/journal.pone.0352865)
Supplement: Peer review history

# Peer review: Decision Letter 0

**DOI:** 10.1371/journal.pone.0352865.r001

**Authors:** Vijay Gc

13 Jan 2026

PONE-D-25-55661

Cost-effectiveness of a mentalisation-based treatment for antisocial personality disorder in males convicted of an offence on community probation in England and Wales: multicentre, assessor-blind randomised controlled trial

PLOS One

Dear Dr. Barrett,

Thank you for submitting your manuscript to PLOS ONE. After careful consideration, we feel that it has merit but does not fully meet PLOS ONE’s publication criteria as it currently stands. Therefore, we invite you to submit a revised version of the manuscript that addresses the points raised during the review process.

Please submit your revised manuscript by Feb 27 2026 11:59PM. If you will need more time than this to complete your revisions, please reply to this message or contact the journal office at plosone@plos.org. When you're ready to submit your revision, log on to https://www.editorialmanager.com/pone/ and select the 'Submissions Needing Revision' folder to locate your manuscript file.

Please include the following items when submitting your revised manuscript:

- A letter that responds to each point raised by the academic editor and reviewer(s). You should upload this letter as a separate file labeled 'Response to Reviewers'.
- A marked-up copy of your manuscript that highlights changes made to the original version. You should upload this as a separate file labeled 'Revised Manuscript with Track Changes'.
- An unmarked version of your revised paper without tracked changes. You should upload this as a separate file labeled 'Manuscript'.

If you would like to make changes to your financial disclosure, please include your updated statement in your cover letter. Guidelines for resubmitting your figure files are available below the reviewer comments at the end of this letter.

If applicable, we recommend that you deposit your laboratory protocols in protocols.io to enhance the reproducibility of your results. Protocols.io assigns your protocol its own identifier (DOI) so that it can be cited independently in the future. For instructions see: https://journals.plos.org/plosone/s/submission-guidelines#loc-laboratory-protocols. Additionally, PLOS ONE offers an option for publishing peer-reviewed Lab Protocol articles, which describe protocols hosted on protocols.io. Read more information on sharing protocols at https://plos.org/protocols?utm_medium=editorial-email&utm_source=authorletters&utm_campaign=protocols.

We look forward to receiving your revised manuscript.

Kind regards,

Vijay S. Gc, PhD

Academic Editor

PLOS One

Journal Requirements:

When submitting your revision, we need you to address these additional requirements.

1. Please ensure that your manuscript meets PLOS ONE's style requirements, including those for file naming. The PLOS ONE style templates can be found at

https://journals.plos.org/plosone/s/file?id=wjVg/PLOSOne_formatting_sample_main_body.pdf and

https://journals.plos.org/plosone/s/file?id=ba62/PLOSOne_formatting_sample_title_authors_affiliations.pdf

2. Thank you for stating the following in the Acknowledgments Section of your manuscript:

“This study was funded by the NIHR Health Technology Assessment Programme (14/186/01). The views expressed are those of the authors and not necessarily those of the UK National Health Service, the NIHR, or the UK Department of Health. PF had full access to all the study data after the database had been approved and locked by the Clinical Trials Unit and had final responsibility for the decision to submit for publication. We would like to thank the research assistants and peer researchers involved in acquiring the data across the 13 sites and the MBT site team leads, clinicians, specialist offender managers, and assistant psychologists for all their hard work with screening and recruiting participants for the RCT and delivering the MBT service; Sarah Skett, Nick Joseph, and Carine Lewis for their guidance and support with implementing an RCT in the criminal justice system and accessing the offending data; The London Probation Pathways and Penrose for helping us identify service user experts for consultation during the feasibility phase of the study; User Voice for their support in collaborating on this trial, for their contribution to peer-led research, and their unwavering commitment to optimal service user involvement; Conor Duggan and the members of the Trial Steering Committee he chaired; and Philip Graham and the members of the Data Management and Ethics Committee he chaired, for their counsel.”

We note that you have provided additional information within the Acknowledgements Section that is not currently declared in your Funding Statement. Please note that funding information should not appear in the Acknowledgments section or other areas of your manuscript. We will only publish funding information present in the Funding Statement section of the online submission form.

Please remove any funding-related text from the manuscript and let us know how you would like to update your Funding Statement. Currently, your Funding Statement reads as follows:

“This work was supported by National Institute for Health Research Health Technology Assessment programme award id 14/186/01.”

Please include your amended statements within your cover letter; we will change the online submission form on your behalf.

3. Thank you for stating the following financial disclosure:

“This work was supported by National Institute for Health Research Health Technology Assessment programme award id 14/186/01.”

Please state what role the funders took in the study.  If the funders had no role, please state: "The funders had no role in study design, data collection and analysis, decision to publish, or preparation of the manuscript."

If this statement is not correct you must amend it as needed.

Please include this amended Role of Funder statement in your cover letter; we will change the online submission form on your behalf.

4. Thank you for stating the following in the Competing Interests section:

“PF and AB report receipt of honoraria for MBT training.”

Please confirm that this does not alter your adherence to all PLOS ONE policies on sharing data and materials, by including the following statement: "This does not alter our adherence to  PLOS ONE policies on sharing data and materials.” (as detailed online in our guide for authors http://journals.plos.org/plosone/s/competing-interests).  If there are restrictions on sharing of data and/or materials, please state these. Please note that we cannot proceed with consideration of your article until this information has been declared.

Please include your updated Competing Interests statement in your cover letter; we will change the online submission form on your behalf.

5. If the reviewer comments include a recommendation to cite specific previously published works, please review and evaluate these publications to determine whether they are relevant and should be cited. There is no requirement to cite these works unless the editor has indicated otherwise.

Additional Editor Comments:

Please submit the completed CHEERS checklist.

[Note: HTML markup is below. Please do not edit.]

Reviewers' comments:

Reviewer's Responses to Questions

**Comments to the Author**

1. Is the manuscript technically sound, and do the data support the conclusions?

The manuscript must describe a technically sound piece of scientific research with data that supports the conclusions. Experiments must have been conducted rigorously, with appropriate controls, replication, and sample sizes. The conclusions must be drawn appropriately based on the data presented.

Reviewer #1: Yes

Reviewer #2: Partly

**********

2. Has the statistical analysis been performed appropriately and rigorously?

Reviewer #1: Yes

Reviewer #2: Yes

**********

3. Have the authors made all data underlying the findings in their manuscript fully available?

The PLOS Data policy requires authors to make all data underlying the findings described in their manuscript fully available without restriction, with rare exception (please refer to the Data Availability Statement in the manuscript PDF file). The data should be provided as part of the manuscript or its supporting information, or deposited to a public repository. For example, in addition to summary statistics, the data points behind means, medians and variance measures should be available. If there are restrictions on publicly sharing data—e.g. participant privacy or use of data from a third party—those must be specified.

Reviewer #1: Yes

Reviewer #2: Yes

**********

4. Is the manuscript presented in an intelligible fashion and written in standard English?

PLOS ONE does not copyedit accepted manuscripts, so the language in submitted articles must be clear, correct, and unambiguous. Any typographical or grammatical errors should be corrected at revision, so please note any specific errors here.

Reviewer #1: Yes

Reviewer #2: Yes

**********

5. Review Comments to the Author

Please use the space provided to explain your answers to the questions above. You may also include additional comments for the author, including concerns about dual publication, research ethics, or publication ethics. (Please upload your review as an attachment if it exceeds 20,000 characters)

Reviewer #1: This manuscript is concise, generally well-written and compelling. The authors describe a cost-effectiveness comparison of probation vs. probation + MBT for men with antisocial personality disorder. They temper their conclusion appropriately given study limitations but suggest that MBT may reduce aggressive behavior and save money. I have a few concerns that are likely addressable via revision.

1. The authors note that they developed the SF-SUS developed for this study. Given the novelty of this measure, I find the lack of psychometric data unsatisfying.

2. Authors report “no systematic pattern in the missing data” but also report that those with complete data were older. Is there no concern that this older sample might be less likely to recidivate or less aggressive? If using their values to predict and impute missing values, might this systematically skew the results?

3. The authors indicate “a cost saving of £92.69 per one-point reduction in OAS-M score.” I’d like to see more interpretation of this finding – how meaningful is a one-point reduction on the OAS-M?

Reviewer #2: I’ve read with interest the manuscript “Cost-effectiveness of a mentalisation-based treatment for antisocial personality disorder in males convicted of an offence on community probation in England and Wales : multicentre assessor blind randomised controlled trial. It was nice to read and overall the economic evaluation has been conducted well although some additional analyses need to be added and/or better explained. Please find my remarks below:

Methods:

• The authors mention the Secure Service Use Schedule tool which is a self-reported tool and administered via structured interviews by researchers.

1) What exactly does this tool measure? Please explain this a bit more for readers that are not familiar with the tool.

• Why were 30 datasets used for multiple imputation? Given that the percentage of missing data exceeds 50%, could you provide a rationale for choosing 30 imputations.

• Lines 151–153: you state that regression analysis was used to compare costs, but it is unclear which type of regression analysis was applied. In addition, based on Table 5, it appears that you also adjusted for baseline utility, but this is not mentioned here. Personally, I am used to applying a seemingly unrelated regression (SUR) to both costs and utility and subsequently using this as input for the economic evaluation. However, it is unclear what exact approach was used in your analysis. Please clarify more.

• The authors present costs and utility scores separately based on complete-case analyses and then only use the multiple imputation (MI) dataset when combining the two outcomes. My suggestion would be to present costs and utility scores based on the MI dataset, as this is standard practice. One option would be to subsequently perform the cost-effectiveness analysis on complete cases and present this as a scenario analysis. This would allow you to discuss the potential impact of using MI versus complete-case analyses on the results and conclusions.

• It is unclear which variables were included in the imputation model ( like age or other patient characteristics?) Could you please add this info?

• It is also unclear which software was used for the analysis? Did they use R ( a useful tutorial for a trial based economic evaluation has been published. See: https://doi.org/10.1007/s40273-023-01301-7) or Stata combined with excell? Please specify this in your method section.

Results

• The ICER based on the OAS-M scores indicates dominance, i.e. lower costs and higher effectiveness. The ICER based on QALYs indicates cost savings but with a QALY loss. I would describe the results explicitly in this way, as this accurately reflects your findings. I am not sure why this result is described as difficult to interpret, as it is essentially the mirror image of the quadrant where additional costs are incurred for a QALY gain. The main issue is that a threshold is typically defined for that quadrant but not for the opposite one. For the south-west quadrant, the relevant question is therefore: what do we consider to be minimally acceptable cost savings for a QALY loss? This could be addressed in the Discussion. Regardless of whether cost savings per QALY lost or a net benefit approach is used, it always requires a discussion of what level of cost savings might be considered acceptable.

• In addition, given that 56% of the bootstrapped cost–effectiveness pairs lie in the quadrant showing cost savings with a QALY loss, and 33% lie in the north-west quadrant where there are higher costs and a QALY loss (i.e. the intervention is inferior), it is unclear what the willingness-to-pay for a QALY represents in Figure 2. What exactly is being paid for, or should this be interpreted as a willingness to accept rather than a willingness to pay?

• Although I understand why the description of the cost-effectiveness plane in Figure 3 was changed, this has become confusing. By switching the axes , it is still not immediately clear that the dominant quadrant reflects an intervention that is more effective and less costly. I am not sure how this could best be resolved. One option might be to only present the cost-effectiveness acceptability curve, because from that figure (please replace “XX” with the figure number) it becomes clear that even under an extreme scenario in which decision-makers are not willing to invest anything, the intervention still has a high probability of being cost-effective.

• Lines 215–217: you mention a sensitivity analysis, but the results are not presented. These should be reported. In addition, it might be useful to add a scenario analysis that only includes healthcare costs, in order to assess the impact of adopting a different analytical perspective.

Discussion

• Returning to the interpretation of the results, particularly in line 239: what does it mean that the cost savings exceed plausible thresholds for willingness to pay for a small QALY loss? Why would society ever be willing to pay for a loss in QALYs, or do you mean something else here? This point needs clarification.

• For the remainder of the Discussion, the authors appropriately describe the limitations of the study and discuss the known issues associated with using a questionnaire such as the EQ-5D-5L in this population. I therefore have no further comments on this section. However, if the additional scenario and sensitivity analyses are conducted, a brief discussion of their implications would be appropriate.

**********

6. PLOS authors have the option to publish the peer review history of their article (what does this mean?). If published, this will include your full peer review and any attached files.

If you choose “no”, your identity will remain anonymous but your review may still be made public.

**Do you want your identity to be public for this peer review?**  For information about this choice, including consent withdrawal, please see our Privacy Policy.

Reviewer #1: No

Reviewer #2: No

**********

[NOTE: If reviewer comments were submitted as an attachment file, they will be attached to this email and accessible via the submission site. Please log into your account, locate the manuscript record, and check for the action link "View Attachments". If this link does not appear, there are no attachment files.]

To ensure your figures meet our technical requirements, please review our figure guidelines: https://journals.plos.org/plosone/s/figures

You may also use PLOS’s free figure tool, NAAS, to help you prepare publication quality figures: https://journals.plos.org/plosone/s/figures#loc-tools-for-figure-preparation.

NAAS will assess whether your figures meet our technical requirements by comparing each figure against our figure specifications.

# Author comment: Author response to Decision Letter 1

**DOI:** 10.1371/journal.pone.0352865.r002

21 May 2026

Review Comments to the Author

Reviewer #1: This manuscript is concise, generally well-written and compelling. The authors describe a cost-effectiveness comparison of probation vs. probation + MBT for men with antisocial personality disorder. They temper their conclusion appropriately given study limitations but suggest that MBT may reduce aggressive behavior and save money. I have a few concerns that are likely addressable via revision.

1. The authors note that they developed the SF-SUS developed for this study. Given the novelty of this measure, I find the lack of psychometric data unsatisfying.

Thank you for raising this point. Many measurement tools undergo psychometric validation; however, service use schedules used in economic evaluations differ conceptually from psychological scales and they are therefore typically validated through developmental refinement and extensive field use, which has been the case with The Secure Facilities Service Use Schedule (SF SUS). was adapted for this study from the original Service Use Schedule, which has been used widely in economic evaluations across health and forensic settings. Its validity rests on extensive previous use in large clinical and forensic trials, iterative development and expert feedback, and alignment with established costing frameworks used in UK health economics. This approach is entirely consistent with best practice guidance for economic evaluations in pragmatic trials. To clarify this point, we have added text in the Methods.

2. Authors report “no systematic pattern in the missing data” but also report that those with complete data were older. Is there no concern that this older sample might be less likely to recidivate or less aggressive? If using their values to predict and impute missing values, might this systematically skew the results?

Thank you for raising this important point. Our statement that there was “no systematic pattern” refers to the fact that missingness did not vary meaningfully by trial arm and was not associated with baseline aggression or other key outcome related variables. The slight age difference you note does not contradict the assumption that the data were missing at random (MAR). We included age in the imputation model, the pattern of missingness was balanced across trial arms, and the findings were consistent across complete case, MI, and sensitivity analyses.

We have now clarified this point in the revised manuscript by explaining that observed predictors of missingness (including age) were explicitly modelled in the imputation procedure, thereby minimising the potential for systematic bias.

3. The authors indicate “a cost saving of £92.69 per one-point reduction in OAS-M score.” I’d like to see more interpretation of this finding – how meaningful is a one-point reduction on the OAS-M?

We agree that a one-point change on the OAS-M does not have a direct clinical interpretation. We included this CEA as a complementary analysis to the main CUA, and to provide a broader picture of how cost differences relate to outcome measures. We have revised the manuscript to clarify that the cost-effectiveness analysis is best interpreted as an indicator of direction and dominance rather than as a precise valuation of a unit of change.

Reviewer #2: I’ve read with interest the manuscript “Cost-effectiveness of a mentalisation-based treatment for antisocial personality disorder in males convicted of an offence on community probation in England and Wales : multicentre assessor blind randomised controlled trial. It was nice to read and overall the economic evaluation has been conducted well although some additional analyses need to be added and/or better explained. Please find my remarks below:

Methods:

• The authors mention the Secure Service Use Schedule tool which is a self-reported tool and administered via structured interviews by researchers.

1) What exactly does this tool measure? Please explain this a bit more for readers that are not familiar with the tool.

Thank you for this helpful suggestion, we have amended the manuscript to describe the different types of service use covered by the SF=SUS.

• Why were 30 datasets used for multiple imputation? Given that the percentage of missing data exceeds 50%, could you provide a rationale for choosing 30 imputations.

Thanks for identifying this, the reviewer is correct that the guidance states that the number of imputed datasets should be similar to the percentage of incomplete cases (Faria, 2014) and therefore we have re-run the MI with 50 imputations. The methods section of the paper has been amended to reflect this change, and the results show the revised MI.

• Lines 151–153: you state that regression analysis was used to compare costs, but it is unclear which type of regression analysis was applied. In addition, based on Table 5, it appears that you also adjusted for baseline utility, but this is not mentioned here. Personally, I am used to applying a seemingly unrelated regression (SUR) to both costs and utility and subsequently using this as input for the economic evaluation. However, it is unclear what exact approach was used in your analysis. Please clarify more.

We have clarified that we used linear regression here and noted that baseline utility was included in the adjustment terms.

• The authors present costs and utility scores separately based on complete-case analyses and then only use the multiple imputation (MI) dataset when combining the two outcomes. My suggestion would be to present costs and utility scores based on the MI dataset, as this is standard practice. One option would be to subsequently perform the cost-effectiveness analysis on complete cases and present this as a scenario analysis. This would allow you to discuss the potential impact of using MI versus complete-case analyses on the results and conclusions.

We thank the reviewer for this thoughtful suggestion. Our decision to present cost and utility scores based on complete data, with MI for the cost-utility and cost-effectiveness analysis, was deliberate. The complete case tables for costs and utility scores are intended to be descriptive, allowing readers to see the observed data that underpin the analysis. We hope that this maintains transparency in our presentation of the trial data. We fully agree that MI is appropriate for the estimation of incremental costs and outcomes, where bias due to missingness is of greatest consequence.

We have amended the manuscript to explain our rationale more clearly.

• It is unclear which variables were included in the imputation model ( like age or other patient characteristics?) Could you please add this info?

Thanks for the suggestion, this has been added.

• It is also unclear which software was used for the analysis? Did they use R ( a useful tutorial for a trial based economic evaluation has been published. See: https://doi.org/10.1007/s40273-023-01301-7) or Stata combined with excell? Please specify this in your method section.

Thanks for the suggestion, this has been added.

Results

• The ICER based on the OAS-M scores indicates dominance, i.e. lower costs and higher effectiveness. The ICER based on QALYs indicates cost savings but with a QALY loss. I would describe the results explicitly in this way, as this accurately reflects your findings.

I am not sure why this result is described as difficult to interpret, as it is essentially the mirror image of the quadrant where additional costs are incurred for a QALY gain. The main issue is that a threshold is typically defined for that quadrant but not for the opposite one. For the south-west quadrant, the relevant question is therefore: what do we consider to be minimally acceptable cost savings for a QALY loss? This could be addressed in the Discussion.

Regardless of whether cost savings per QALY lost or a net benefit approach is used, it always requires a discussion of what level of cost savings might be considered acceptable.

Thank you for this helpful and constructive suggestion, we agree that the results can be described more explicitly and consistently. We have revised the manuscript to clearly state that the OAS-M based CEA indicates dominance and the QALY based analysis indicates lower costs accompanied by a small QALY loss. Indeed the challenge with the QALY result is not technical (we have removed this) but the absence of an explicit decision threshold for the south west quadrant. We have therefore expanded the discussion section to explicit state this and consider the implications.

• In addition, given that 56% of the bootstrapped cost–effectiveness pairs lie in the quadrant showing cost savings with a QALY loss, and 33% lie in the north-west quadrant where there are higher costs and a QALY loss (i.e. the intervention is inferior), it is unclear what the willingness-to-pay for a QALY represents in Figure 2. What exactly is being paid for, or should this be interpreted as a willingness to accept rather than a willingness to pay?

We thank the reviewer for this important observation and agree that the interpretation of the WTP requires clarification. We have revised the text accompanying figure 2 to make it clear that the CEAC illustrates the probability of cost-effectiveness for different values a decision maker is willing to accept in terms of cost savings for small losses in quality of life.

• Although I understand why the description of the cost-effectiveness plane in Figure 3 was changed, this has become confusing. By switching the axes , it is still not immediately clear that the dominant quadrant reflects an intervention that is more effective and less costly. I am not sure how this could best be resolved. One option might be to only present the cost-effectiveness acceptability curve, because from that figure (please replace “XX” with the figure number) it becomes clear that even under an extreme scenario in which decision-makers are not willing to invest anything, the intervention still has a high probability of being cost-effective.

Thank you for highlighting this issue; we agree that reversing the axis for the OAS-M based CE plane, whilst analytically correct, may result in confusion. We would prefer to retain figure 3, since it provides a visual representation of the joint distribution of the cost and outcomes and is consistent with the QALY results. We hope to mitigate this by more explicit labelling of quadrants and the figure itself, and cross referencing to the CEAC.

• Lines 215–217: you mention a sensitivity analysis, but the results are not presented. These should be reported. In addition, it might be useful to add a scenario analysis that only includes healthcare costs, in order to assess the impact of adopting a different analytical perspective.

We have added in the sensitivity analyses to the results section, and included a service perspective which includes health, social care, and criminal justice costs. We fell that given the setting and the patient group this is a more appropriate perspective that just a health care alone.

Discussion

• Returning to the interpretation of the results, particularly in line 239: what does it mean that the cost savings exceed plausible thresholds for willingness to pay for a small QALY loss? Why would society ever be willing to pay for a loss in QALYs, or do you mean something else here? This point needs clarification.

Thank you for identifying this point of potential confusion, we have removed it as (thanks to your earlier comment) we hope this section is now better explained.

• For the remainder of the Discussion, the authors appropriately describe the limitations of the study and discuss the known issues associated with using a questionnaire such as the EQ-5D-5L in this population. I therefore have no further comments on this section. However, if the additional scenario and sensitivity analyses are conducted, a brief discussion of their implications would be appropriate.

Thanks for all your very helpful comments. Since the (now) reported sensitivity analyses did not alter the direction or magnitude of the cost differences, and that they are consistent with those already mentioned in the manuscript, we don’t think it’s necessary to add anything additional to the discussion section.

Attachment Submitted filename: moam cea plos one response to reviewers 29012026.docx

# Peer review: Decision Letter 1

**DOI:** 10.1371/journal.pone.0352865.r003

**Authors:** Vijay Gc, Vijay Gc

16 Jun 2026

Cost-effectiveness of a mentalisation-based treatment for antisocial personality disorder in males convicted of an offence on community probation in England and Wales: multicentre, assessor-blind randomised controlled trial

PONE-D-25-55661R1

Dear Dr. Barrett,

We’re pleased to inform you that your manuscript has been judged scientifically suitable for publication and will be formally accepted for publication once it meets all outstanding technical requirements.

Within one week, you’ll receive an e-mail detailing the required amendments. When these have been addressed, you’ll receive a formal acceptance letter and your manuscript will be scheduled for publication.

An invoice will be generated when your article is formally accepted. Please note, if your institution has a publishing partnership with PLOS and your article meets the relevant criteria, all or part of your publication costs will be covered. Please make sure your user information is up-to-date by logging into Editorial Manager at Editorial Manager® and clicking the ‘Update My Information' link at the top of the page. For questions related to billing, please contact billing support.

If your institution or institutions have a press office, please notify them about your upcoming paper to help maximize its impact. If they’ll be preparing press materials, please inform our press team as soon as possible -- no later than 48 hours after receiving the formal acceptance. Your manuscript will remain under strict press embargo until 2 pm Eastern Time on the date of publication. For more information, please contact onepress@plos.org.

Kind regards,

Vijay S. Gc, PhD

Academic Editor

PLOS One

Additional Editor Comments (optional):

Reviewers' comments:

Reviewer's Responses to Questions

**Comments to the Author**

1. If the authors have adequately addressed your comments raised in a previous round of review and you feel that this manuscript is now acceptable for publication, you may indicate that here to bypass the “Comments to the Author” section, enter your conflict of interest statement in the “Confidential to Editor” section, and submit your "Accept" recommendation.

Reviewer #1: All comments have been addressed

Reviewer #2: All comments have been addressed

**********

2. Is the manuscript technically sound, and do the data support the conclusions?

The manuscript must describe a technically sound piece of scientific research with data that supports the conclusions. Experiments must have been conducted rigorously, with appropriate controls, replication, and sample sizes. The conclusions must be drawn appropriately based on the data presented.

Reviewer #1: Yes

Reviewer #2: (No Response)

**********

3. Has the statistical analysis been performed appropriately and rigorously?

Reviewer #1: Yes

Reviewer #2: (No Response)

**********

4. Have the authors made all data underlying the findings in their manuscript fully available?

The PLOS Data policy requires authors to make all data underlying the findings described in their manuscript fully available without restriction, with rare exception (please refer to the Data Availability Statement in the manuscript PDF file). The data should be provided as part of the manuscript or its supporting information, or deposited to a public repository. For example, in addition to summary statistics, the data points behind means, medians and variance measures should be available. If there are restrictions on publicly sharing data—e.g. participant privacy or use of data from a third party—those must be specified.

Reviewer #1: Yes

Reviewer #2: (No Response)

**********

5. Is the manuscript presented in an intelligible fashion and written in standard English?

PLOS ONE does not copyedit accepted manuscripts, so the language in submitted articles must be clear, correct, and unambiguous. Any typographical or grammatical errors should be corrected at revision, so please note any specific errors here.

Reviewer #1: Yes

Reviewer #2: (No Response)

**********

6. Review Comments to the Author

Please use the space provided to explain your answers to the questions above. You may also include additional comments for the author, including concerns about dual publication, research ethics, or publication ethics. (Please upload your review as an attachment if it exceeds 20,000 characters)

Reviewer #1: (No Response)

Reviewer #2: (No Response)

**********

7. PLOS authors have the option to publish the peer review history of their article (what does this mean?). If published, this will include your full peer review and any attached files.

If you choose “no”, your identity will remain anonymous but your review may still be made public.

**Do you want your identity to be public for this peer review?**  For information about this choice, including consent withdrawal, please see our Privacy Policy.

Reviewer #1: No

Reviewer #2: No

**********

# Editor report: Acceptance letter

**DOI:** 10.1371/journal.pone.0352865.r004

**Authors:** Vijay Gc, Vijay Gc

PONE-D-25-55661R1

PLOS One

Dear Dr. Barrett,

I'm pleased to inform you that your manuscript has been deemed suitable for publication in PLOS One. Congratulations! Your manuscript is now being handed over to our production team.

At this stage, our production department will prepare your paper for publication. This includes ensuring the following:

* All references, tables, and figures are properly cited

* All relevant supporting information is included in the manuscript submission,

* There are no issues that prevent the paper from being properly typeset

You will receive further instructions from the production team, including instructions on how to review your proof when it is ready. Please keep in mind that we are working through a large volume of accepted articles, so please give us a few days to review your paper and let you know the next and final steps.

Lastly, if your institution or institutions have a press office, please let them know about your upcoming paper now to help maximize its impact. If they'll be preparing press materials, please inform our press team within the next 48 hours. Your manuscript will remain under strict press embargo until 2 pm Eastern Time on the date of publication. For more information, please contact onepress@plos.org.

You will receive an invoice from PLOS for your publication fee after your manuscript has reached the completed accept phase. If you receive an email requesting payment before acceptance or for any other service, this may be a phishing scheme. Learn how to identify phishing emails and protect your accounts at https://explore.plos.org/phishing.

If we can help with anything else, please email us at customercare@plos.org.

Thank you for submitting your work to PLOS ONE and supporting open access.

Kind regards,

PLOS ONE Editorial Office Staff

on behalf of

Dr. Vijay S. Gc

Academic Editor

PLOS One
